# Supplementary material for: Constructing a Low–Cost Si–NSs@C/NG Composite by a Ball Milling–Catalytic Pyrolysis Method for Lithium Storage
Source: Molecules. 2023 Apr 14;28(8):3458. doi: 10.3390/molecules28083458 (PMC10145678; doi:10.3390/molecules28083458)
Supplement: Supplementary file 1 [file molecules-28-03458-s001.zip › molecules-2315193-supplementary.pdf]

Supporting Information

Constructing Low-cost Si-NSs@C/NG Composite by Ball milling-catalytic Pyrolysis for Lithium Storage

Qi Zhang, Ning-Jing Song, Canliang Ma, Yun Zhao, Yong Li, Juan Li，Xiaoming Li, Qingqiang Kong, Cheng-meng Chen

Table S1. Detailed calculation process of the yield of ($NG+C+Fe_2O_3$) in products.

Samples	Raw materials	Products	The yield of $NG+C+Fe_2O_3$ in products
NG	3g C_3N_4+0.2g ferrocene	0.0318g (NG +C +Fe_2O_3)	0.0318/3.2* 100%=0.99%
Si-NSs@C/NG	3g C_3N_4+0.1gSi-NSs +0.2g ferrocene	0.2926g (NG+C +Fe_2O_3+Si-NSs)	(0.2926-0.1)/3.2* 100%=6.02%

Note: The mass of Si-NSs remained the same before and after the reaction.

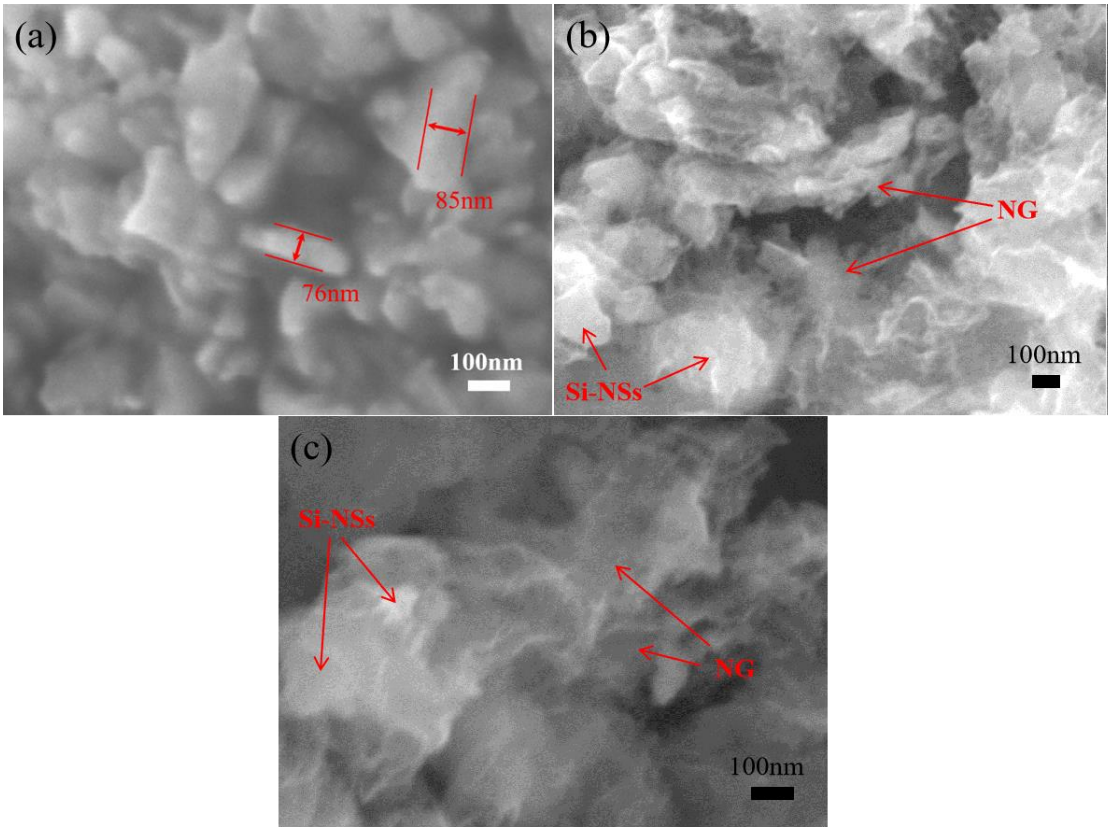

Figure S1. SEM images of Si-NSs(a) and Si-NSs@C/NG(b , c)

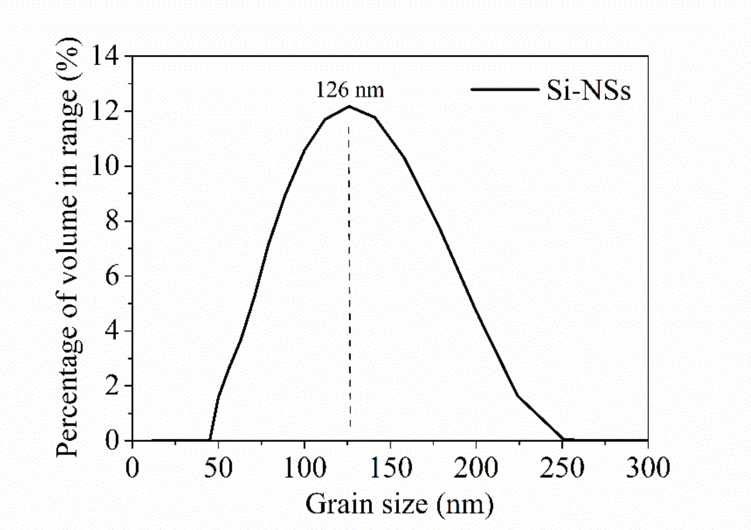

Figure S2. Lateral size distribution of Si-NSs.

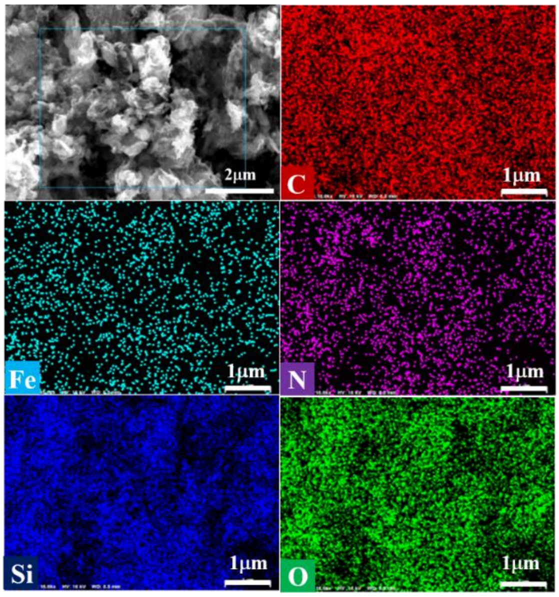

Figure S3. Elemental mapping images of Si-NSs@C/NG.

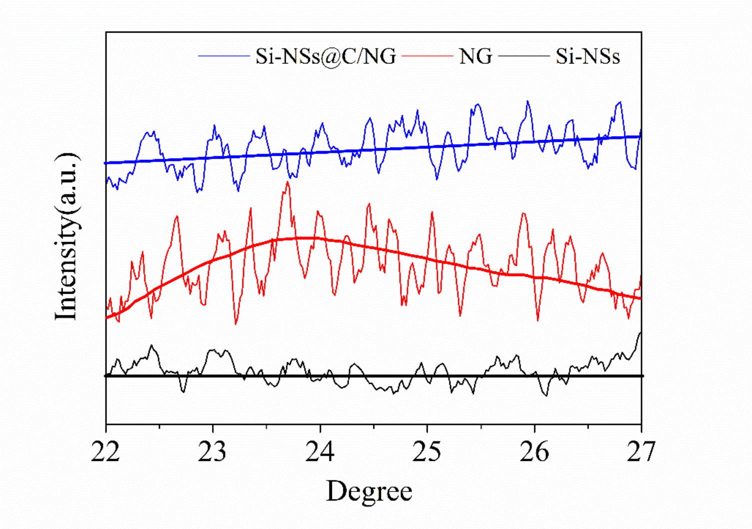

Figure S4. XRD patterns of Si-NSs, NG and Si-NSs@C/NG.

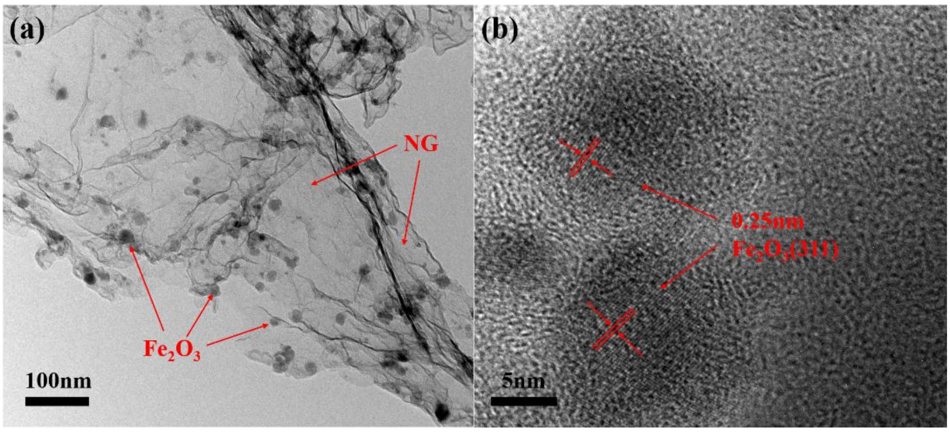

Figure S5. TEM images of NG.

Table S2.The element components in the samples determined by XPS analysis (at.%).

Samples	C	N	O	Fe	Si
Si-NSs@C/NG	58.12	11.64	22.41	1.32	6.52
